# Methods for Translating Evidence-Based Behavioral Interventions for Health-Disparity Communities

**DOI:** 10.5888/pcd10.130133

**Published:** 2013-11-21

**Authors:** Anna Maria Nápoles, Jasmine Santoyo-Olsson, Anita L. Stewart

**Affiliations:** Author Affiliations: Jasmine Santoyo-Olsson, Anita L. Stewart, University of California, San Francisco, California.

## Abstract

Populations composed of racial/ethnic minorities, disabled persons, and people with low socioeconomic status have worse health than their counterparts. Implementing evidence-based behavioral interventions (EBIs) to prevent and manage chronic disease and disability in community settings could help ameliorate disparities. Although numerous models of implementation processes are available, they are broad in scope, few offer specific methodological guidance, and few address the special issues in reaching vulnerable populations. Drawing from 2 existing models, we describe 7 methodological phases in the process of translating and implementing EBIs in communities to reach these vulnerable groups: establish infrastructure for translation partnership, identify multiple inputs (information gathering), review and distill information (synthesis), adapt and integrate program components (translation), build general and specific capacity (support system), implement intervention (delivery system), and develop appropriate designs and measures (evaluation). For each phase, we describe specific methodological steps and resources and provide examples from research on racial/ethnic minorities, disabled persons, and those with low socioeconomic status. Our methods focus on how to incorporate adaptations so that programs fit new community contexts, meet the needs of individuals in health-disparity populations, capitalize on scientific evidence, and use and build community assets and resources. A key tenet of our approach is to integrate EBIs with community best practices to the extent possible while building local capacity. We discuss tradeoffs between maintaining fidelity to the EBIs while maximizing fit to the new context. These methods could advance our ability to implement potentially effective interventions to reduce health disparities.

## Introduction

The transfer and application of scientific evidence could help mitigate persistent health disparities (1). Although evidence-based behavioral interventions (EBIs) that prevent disease and disability exist, they are not being implemented broadly, especially in vulnerable communities experiencing disparities (1).

Dissemination and implementation science develops strategies for translating EBIs into real-world settings to promote widespread adoption (2). Numerous conceptual models or frameworks have been developed for dissemination and implementation processes that are applied in clinical settings and health systems (3). However, most are general models or use case studies to illustrate dissemination and implementation processes (4,5). None addresses special issues involved in dissemination and implementation research among individuals in health-disparity communities.

A health-disparity population has significantly higher disease incidence, prevalence, morbidity, or mortality or poorer survival rates than the general population (1) and is generally associated with socioeconomic disadvantages (6). Such populations include racial/ethnic minority groups, people with low socioeconomic status (SES), women, older adults, people with a mental health condition, and people with physical or intellectual disabilities. Particular attention is needed to translate EBIs for individuals with disabilities (7) because they are more likely to have chronic conditions, receive fewer preventive services, experience greater delays in obtaining needed health care, and have higher rates of tobacco use and obesity than their counterparts (8).

Translating EBIs into settings to reach these vulnerable individuals presents numerous unique issues. We focus on the translation of behavioral interventions in community settings because many individuals in health-disparity populations have limited or no access to health care (6). We focus on individual-level behavioral interventions because these play a central role in preventing and managing many prominent health problems that disproportionately affect vulnerable populations (6).

Large differences between the original EBI context and disparity communities in populations, settings, and available resources often require substantial adaptations. Health-disparity communities often have locally developed programs (best practices) that are designed specifically for vulnerable individuals and that warrant consideration (9). These communities often have limited resources; thus, implementation may require community capacity building. Community-based participatory research (CBPR) approaches that create community ownership of EBIs are critical given that these communities experience marginalization.

In this article, we describe specific methodological guidelines for disseminating and implementing EBIs to reach individuals from health-disparity populations for use by researchers working in academic–community partnerships. We describe 7 methodological phases, specific steps within each phase, and examples illustrating unique issues with disparity communities. Because the article focuses on the initial translation into these communities, we do not discuss scale-up or impact on population health, for which other frameworks are more appropriate (eg, RE-AIM) (10).

## Frameworks Used

We draw primarily from 2 published translation frameworks. The Interactive Systems Framework (ISF), developed by the Centers for Disease Control and Prevention, describes 3 nonlinear interactive systems involved in transferring innovations to community settings (11). The synthesis and translation system describes processes to distill and prepare research for dissemination and implementation into practice. This system involves identifying and reviewing EBIs in collaboration with intended audiences to ensure the fit between the EBIs and the new context. The support system involves capacity building to support the specific intervention and community organization that will deliver it. The delivery system involves applying program-specific and general organizational capacities for program implementation in targeted settings. The ISF accommodates varying degrees of academic and community input and recognizes that adaptation may require synthesizing several EBIs and stakeholder knowledge. However, it does not delineate activities for adapting EBIs for use with individuals from racially or ethnically diverse groups or other disparity groups. A framework by Wainberg and colleagues fills this gap, outlining a 4-step adaptation process: 1) optimizing fidelity (eg, identifying EBIs and their common content), 2) optimizing fit (eg, understanding contextual factors and collaborating with community members), 3) balancing fidelity and fit (eg, conducting adaptations), and 4) pilot testing and refining (12).

## Methods for Translating Programs

To develop recommended methods, we integrated and expanded both the ISF and the model by Wainberg and colleagues and classified the types of adaptations needed. We identified 7 iterative, nonlinear implementation phases in health-disparity communities: 1) establish infrastructure for translation partnership; 2) identify multiple inputs (information gathering); 3) review and distill information (synthesis); 4) adapt and integrate candidate program components (translation); 5) build general and specific capacity (support system); 6) implement intervention (delivery system); and 7) develop appropriate design and measures (evaluation). We present the phases in the general order that they occur, although they tend to be iterative as new knowledge is gained. We describe the rationale and special considerations in translating EBIs for populations of racial/ethnic minorities, people with low SES, older people, and disabled persons. We also provide methodological details and examples of health-disparity behavioral interventions in community settings (Table).

**Table:** Methods for Translating Evidence-Based Behavioral Interventions (EBIs) for Health-Disparity Communities

Phase/Recommended Step	Description and Methods	Examples from Translation Research in Health-Disparity Communities
**Phase 1: Establish infrastructure for translation partnership**		
1. Identify partners and secure their meaningful involvement.	Partnerships vary in distribution of power, resources, and decision-making latitude. Partners can include academic researchers, community-based organizations (CBOs), community members, and other stakeholders.	The team for a cognitive-behavioral stress management (CBSM) intervention study for Spanish-speaking cancer patients included researchers, CBOs, clinical sites, community advocates, and a community advisory board (13).
2. Explicitly delineate partners’ roles and responsibilities.	• Create formal memorandum of understanding (MOU), including dispute resolution and data or program ownership policies.	For a community-based pilot study and subsequent randomized controlled trial, MOUs that delineated roles and responsibilities of academic–community partners helped resolve disputes (13).
	• Address hierarchies and issues of inequality in decision making that contribute to disparities (9).	
3. Secure funding for research.	• Obtain institutional seed money.	Separate awards to an academic and community partner to conduct a randomized controlled trial promoted shared responsibility for a study (13).
	• Co-write grant proposals with community partners with shared funding.	
**Phase 2: Identify multiple inputs — information gathering**		
1. Identify multiple EBIs to address specific health disparity.	Identify research-tested behavioral interventions via articles and reviews, meta-analyses, and websites. Secure program materials via websites or by contacting developers.	• National Cancer Institute Research-Tested Intervention Programs: 122 interventions with program materials (14).
		• National Registry of Evidence-Based Programs and Practices: more than 220 mental health and substance abuse interventions (15).
2. Identify community best practices that address specific health disparity.	Identify via community partners, websites, public health planning documents, and stakeholders. Review program rationale and materials; interview program developers or providers.	An academic-community partnership study produced a guide for CBOs documenting best practices for providing cancer support services (13).
3. Collect information on local contextual factors that are related to specific health disparity.	Written and oral narratives, key informant interviews, logs, and inter-organizational network analyses can identify population, organizational, and community factors affecting intervention uptake, success, and sustainability (16).	A study of people with disabilities using mobility devices found that the extent and type of mobile technology used depended on the physical context and available types of support (17).
4. Conduct formative research with community stakeholders.	Formative research methods include key informant interviews, focus groups, community forums, and field observations to understand disparities and their determinants (12,13,18).	Focus group and individual interviews with community members, administrators, health care providers, and physical activity instructors identified issues in providing physical activity programs to reduce disparities (19).
**Phase 3: Review and distill information — synthesis**		
1. Consider how EBIs, community best practices, and formative research results can be integrated into a potential intervention.	Identify “active ingredients” of candidate EBIs and best practices. EBIs usually share key core components that can be reviewed and synthesized (12,20). Examine fit of best practices to active ingredients of EBIs.	Peers were trained to deliver a cognitive-behavioral stress-management intervention for Spanish-speaking cancer patients (13).
2. Review potential intervention components to determine fit to contextual factors including population, delivery system, and community context characteristics.	• Build consensus through meetings and forums on fit of potential intervention components and potential to address disparities.	• Partnership members reviewed several iterations of a CBSM program to determine fit to Spanish-speaking cancer patients and community delivery channels (13).
	• Consider relevant population characteristics (culture, literacy, language, preferred learning channels, socioeconomic status, and disabilities) in design of content, messages, and format; identify missing content.	• Universal Design of Research principles describe environmental supports that promote inclusion of persons with disabilities in intervention research (21).
	• Review organizational structure, staff, skills, and interorganizational networks within which agencies operate to deliver interventions.	• To ensure that components could be integrated into practice, community practitioners were involved in translating a caregiver support intervention (5).
**Phase 4: Adapt and integrate program components — translation**		
1. Based on synthesis process, design specific adaptations that will be needed.	Select targeted adaptations and designate which team members are responsible for specific tasks.	Academic and community partners worked together to perform adaptations and language translation of materials (13).
1a. Adapt to population culture and language.	Several methodological frameworks for culturally adapted interventions have been described (18,22).	Cultural adaptations to an evidence-based CBSM program for Spanish-speaking cancer patients included emphasis on asking for help, communicating with physicians, identifying resources, and role of family interdependency (13).
1b. Adapt to population literacy and preferred channels for sharing of information, making materials accessible and user friendly and facilitating data collection.	• Detailed guidebooks for preparation of low-literacy materials are available (23).	For self-management interventions, using technology to allow visual, audible, and tactile output (eg, talking pedometers, blood glucose meters with large-print readouts) provided options for disabled populations (21).
	• Factors that need to be considered are reading level, cultural beliefs, making materials interactive, use of visuals, messages that are supportive of racial/ethnic practices, use of concrete examples, and providing how-to information (21).	
	• Because preferred communication channels can vary by race/ethnicity, geography, disability, and socioeconomic status, review literature and results of public health campaigns to identify options (24).	
1c. Adapt for community context.	Using socioecologic models, examine how intervention fits with social, political, and physical environments.	Effective post-stroke rehabilitation depended on ad hoc support by family and social network members who provided opportunities for physical activities (25).
1d. Adapt for specific vulnerabilities of targeted individuals.	Tailor program to daily living conditions, resource limitations, and other vulnerabilities that characterize a disparity population. Common barriers include limited access to services, discrimination experiences, and transportation difficulties.	Because of participants’ economic hardship, information on community resources (eg, financial support, housing, transportation, social services) was added to a cancer support program (13).
1e. Adapt program delivery methods to enhance sustainability.	• Identify feasible program delivery methods and staffing implications.	• An EBI delivered by health professionals was adapted for delivery by trained community health workers (26).
	• Determine factors affecting sustainability (eg, resource limitations); reach consensus on outcomes to be sustained (11,27).	• To translate a caregiver support program for Area Agencies on Aging, the adapted program contained fewer sessions than the original to reduce cost (4).
2. Integrate adapted components, specify planned intervention, and pretest.	• Document adaptations and rationale, addition or substitution of materials and approaches to fit context; compare form and function of adapted program to original EBIs (28).	Several pretests helped to achieve balance between fidelity and fit of an HIV-prevention intervention among Brazilians with mental health problems (12).
	• Have key stakeholders review intervention or conduct focus groups to pretest intervention; modify as indicated.	
**Phase 5: Build general and specific capacity — support system**		
1. Build community capacity to implement translated program.	Enhance infrastructure and knowledge needed to deliver program successfully. Hire and train community providers to deliver intervention. Create clear and comprehensive operations and intervention manuals.	Translating a physical activity EBI into minority communities required developing new community physical activity resources through interagency collaborations (26).
2. Build community capacity for practical sustainability.	Identify ongoing sources of support for the program and widespread dissemination; build infrastructure for sustainability.	The community-based Chronic Disease Self-Management Program (English and Spanish versions) was embedded within the El Paso Diabetes Association (grantee) to reach Latinos (29).
**Phase 6: Implement intervention — delivery system**		
1. Implement and monitor intervention in community setting.	Create processes and contingency plans for delivery and oversight of program implementation (procedures for delivering intervention, staffing, and accountability). Establish procedures for obtaining feedback; troubleshoot issues with partners.	In an academic-community partnership, the lead community agency received a separate grant to support program implementation by CBOs and to supervise community health workers delivering the program (13).
2. Provide ongoing technical assistance and support.	Track implementation and dissemination processes, challenges, and successes. Make provisions for ongoing technical assistance from program developers, content experts, and community leaders.	An EBI disseminated in African American congregations without researcher or agency involvement did not achieve outcomes comparable to earlier trials, largely because of a lack of ongoing technical assistance to support program implementation (30).
**Phase 7: Develop appropriate design and measures — evaluation**		
1. Develop evaluation designs that are relevant and appropriate for the context.	Implementation science uses a range of evaluation designs. Summary of a symposium sponsored by the National Institutes of Health and Centers for Disease Control and Prevention considered study design choices and tradeoffs for translational research (31).	A translation of an evidence-based dementia-caregiver intervention used a quasiexperimental pre–post treatment design (4).
2. Develop relevant and appropriate measures.	• Process measures include fidelity, reach, time spent on program activities, use of intervention materials, level of participation, dose delivered, external factors, program penetration, program impact, and costs (32).	Measures of community level changes were incorporated in diffusing a physical activity promotion program into minority communities (26).
	• Mixed qualitative and quantitative process evaluation methods can link implementation processes to program and community outcomes (33).	

### Phase 1: Establish infrastructure for translation partnership

Infrastructure is needed to facilitate collaboration and communication among researchers, stakeholders, and community partners. This phase involves 3 steps: 1) identify partners and secure their involvement, 2) explicitly delineate roles and responsibilities, and 3) secure academic-community partnership funding for dissemination and implementation research.

Academic-community collaborations bring together diverse groups to identify practical considerations, mobilize community assets, and ensure that programs address community priorities (34). In health-disparity research, establishing such collaborations is even more important because of the mistrust of research institutions and experiences of discrimination among health-disparity populations. Delineating community and academic partners’ roles and responsibilities enhances communication, expectation management, accountability, and trust. Consensus is reached through the active and meaningful participation of community partners throughout the entire translation process so that the needs and preferences of those who will be affected by the intervention are addressed (33). Reinforcing communication channels is critical in anticipation of the need to troubleshoot challenges that surface during implementation.

### Phase 2: Identify multiple inputs — information gathering

Part of the ISF synthesis system pertains to identifying inputs (eg, evidence and knowledge from multiple sources). We specify 4 steps: 1) identify multiple candidate EBIs, materials, and procedures, 2) identify community best practices, 3) identify contextual factors, and 4) conduct formative research with community stakeholders.

Evidence is defined as core components and constructs from the underlying theoretical model believed to account for an EBI’s effectiveness (11,12). Identifying best practices is equally important in disparity communities because they are designed to meet the needs of vulnerable individuals within existing resources and constraints (33). Such programs integrate front-line knowledge of health disparities and community empowerment. Integrating best practices enhances the potential for scale-up in limited-resource settings (9).

Information about the context for translation helps assess the applicability of research findings beyond controlled clinical settings. Community partners identify community assets to support the program or vulnerabilities that might require capacity building. Formative work can identify local infrastructure that can integrate the targeted program (20).

Formative research with key stakeholders can identify their concerns and preferences (33). Local data on the nature and causes of targeted health disparities inform translation. This is especially critical with underrepresented groups, as data to inform necessary adaptations may be scarce and individuals’ needs may differ substantially from the needs for which the original EBI was developed.

### Phase 3: Review and distill information — synthesis

This complex phase involves having partners review and synthesize inputs while balancing fidelity and fit. We specify 2 steps: 1) review EBIs, community best practices, and formative research results in terms of how they can be integrated into an intervention, and 2) consider how components can accommodate population characteristics, delivery system, and community context. These steps are done simultaneously to narrow down the set of EBIs that are feasible and incorporate community assets (34).

Identifying differences between target groups and original EBI samples helps gauge intervention components’ potential acceptability, effectiveness, and reach. Candidate program components are reviewed for their match to the intended delivery system. Program delivery venues available in disparity communities will likely have fewer resources than the original EBI settings; for example, lay health workers rather than professionals may need to deliver the program. Synthesis of distinct theoretical or practical approaches may occur.

Academic and community partners review community-level resources that might affect program adoption such as communication channels, political and built environments, transportation, competing programs, social networks, community and practice norms, and neighborhood living conditions. Knowledge of the community facilitates identification of local resources that can be tapped or capacities that need to be developed (9).

The synthesis process occurs with partnership members selecting the optimal features of all inputs for potential inclusion. Areas needing adaptations to accommodate differences in characteristics of targeted individuals, delivery system, and community context from the original EBI are identified. In addressing disparities, the scope of needed adaptations will be greater than in nondisparity populations because EBIs are rarely designed for vulnerable groups.

### Phase 4: Adapt and integrate program components — translation

Adaptation refers to processes of changing an intervention to suit the needs and characteristics of a setting (eg, population) (35). This phase entails making adaptations to generate a program prototype and involves 2 steps: 1) design needed adaptations, and 2) create and pretest program prototype.

Health disparities research requires adaptations to accommodate factors related to culture, language, literacy, preferred channels for receiving information, community context, individuals’ vulnerabilities, and delivery methods. It is helpful to document tradeoffs made between feasibility, fidelity, and acceptability to build evidence of optimal translation methods. We discuss 5 types of adaptations.

**Culture and language**. Cultural and linguistic adaptations result in greater effectiveness and relevance to racially and ethnically diverse groups (36). We classify cultural adaptations as the following: 1) change the EBI’s key constructs, messages, or methods for increased cultural relevance, 2) incorporate cultural values that reinforce the EBI, 3) add missing content because the original EBIs may not have addressed a group-specific need, 4) identify socio-cultural norms, beliefs, or behaviors that may conflict with EBIs, and 5) identify culturally similar role models to demonstrate the targeted behaviors. Adaptations must balance cultural acceptability of EBI components while maintaining evidence-based active ingredients (ie, fidelity).

**Literacy and preferred learning channels**. Interventions designed for people with limited English proficiency and low levels of literacy require educational approaches because simple health information in their native languages is largely unavailable. Methodological resources for developing health communications for racially or ethnically diverse or low-SES groups recommend identifying the target audience’s characteristics (eg, educational level), presenting information in multiple formats to address various learning styles (ie, visual, aural, kinesthetic, audio-visual, or written), choosing appropriate delivery channels (eg, face-to-face, mass media), and conducting formative research with end users to ensure messages are comprehensible and actionable (24).

**Community context.** Persistent disparities and sustained community health improvements call for application of multilevel ecological models in which community interventions are parts of larger complex systems (10). Partners must consider the structure, processes, and goals of the intervention and how these align with those of the community itself (9). Adaptation of the intervention to the community context involves answering 2 key questions: why is the intervention important to practitioners, and how can practitioners apply the information learned to reduce health disparities (20)? Asking these questions helps identify elements that must be included for program effectiveness and that can be changed to fit the context (20). Adaptation must consider the fundamental culture or defining characteristics of community life (9).

**Specific vulnerabilities**. Tailoring EBIs for individuals in disparity populations requires addressing characteristics (eg, functional limitations, limited resources) that make these individuals more vulnerable than their counterparts. Adaptations to improve accessibility for people with special visual, hearing, mobility, cognitive, emotional, or learning needs maximizes adoption.

**Community-centered program delivery models and sustainability**. Adaptations to program delivery methods need to attend to feasibility and sustainability in community contexts. The most common need is to adapt an intervention to cost less, which typically requires reducing program intensity (4).

Adaptations are implemented through drafts of program manuals and procedures. Because adaptations are likely to be extensive, pretesting is important. Pretests of program components and procedures using mixed methods can identify needed modifications before large trials. After pretesting, academic-community partners refine the final program.

### Phase 5: Build general and specific capacity — support system

This phase acknowledges community capacity building to address health equity (37) and practical issues involved in full-scale community adoption. We specify 2 steps: 1) build capacity of community organizations delivering the intervention, and 2) build community capacity for practical sustainability.

Building capacity entails building community organizations’ general capacity as well as specific resources needed for program delivery and sustainability. Carrying out community implementation depends heavily on prior capacity building to enable those processes (11). Trickett and colleagues (9) define capacities as “resources that can be drawn upon and developed as a function of collaborative research and interventions . . . and may include social participation, interorganizational networks, skills, knowledge, leadership, and social settings.” Methods for building capacity include training, technical assistance, and coaching (20). Building community capacity is consistent with CBPR approaches that augment community strengths to create resources that can be transferred to future health issues (9).

### Phase 6: Implement intervention — delivery system

Phase 6, from the ISF delivery system for ongoing implementation, involves 2 steps: 1) implement and monitor the intervention in community settings, and 2) provide ongoing technical support. Tracking and reporting the multilevel processes necessary to deliver interventions in disparity communities is important to contextualize the study and provide information for gauging the intervention’s external validity (27) for other underserved communities. During this phase, relationships often can become strained if issues of partners’ responsibilities, expectations, and mutual trust have not been addressed adequately in Phase 1. Having processes in place for immediate feedback among collaborators is critical. Incentives for successful implementation need to exist for both academic and community partners. Ongoing technical assistance by program developers and content experts is usually required because of substantial adaptations, less familiarity with research exigencies among communities, and the need to reinforce and balance theoretical underpinnings of the translated program with the new context. Community partners may have ongoing questions on the need for adherence to certain procedures such as randomization and the extent to which those procedures can be modified in practice, which may require dialogue between partners to reach agreement on ways to maintain program and study integrity while optimizing fit.

### Phase 7: Develop appropriate design and measures — evaluation

Numerous issues have been raised about appropriate study designs and measures to evaluate translated interventions in community settings (9,31). Two steps include developing relevant and appropriate designs and measures.

Study design issues relate to the scientific rigor of evaluation designs used in community settings. Whereas evidence supporting the efficacy of EBIs is generated using randomized controlled trials (RCTs), we need to determine whether translated programs are effective in “real world” settings. To assess the effectiveness of programs in community settings, evaluation models other than RCTs are appropriate. RCTs may not be feasible in community settings because investigators have less control over intervention delivery, use of usual-care control groups may be viewed as unethical (31), and resistance to randomization may be heightened in racial/ethnic minority communities. Alternatives to RCTs that are more context-specific have been suggested for evaluating community programs (9,38). Mercer and colleagues (31) explore tradeoffs among various designs applicable to translational research.

Translation researchers recommend evaluating the processes of translation, dissemination, and implementation and developing methods and measures of these processes (33). Process evaluation improves our understanding of mechanisms and moderators (eg, contextual and individual characteristics) of intervention effectiveness and facilitates replication (32). Self-reported individual-level measures must be valid and reliable, and evidence of validity and reliability of certain instruments among diverse populations is often unavailable.

In addition to measuring individual-level outcomes (34) and conducting process evaluation, collecting data on community-level outcomes — such as changes in social networks, resources, knowledge, leadership, and social participation — is useful (9). Saul and colleagues (39) suggest developing measures of general and intervention-specific capacity and changes in individual and organizational practice. Community-level outcomes need to consider the potential effect of reducing disparities; an example of such a community-level outcome is the increased capacity of community health workers to conduct research and health interventions.

## Discussion

We have delineated 7 methodological phases in translating EBIs into vulnerable communities and their specific steps. A key tenet of our approach has been to integrate EBIs with community best practices while building local capacity for addressing disparities. Efforts to adapt and synthesize EBIs with community best practices, even if extensive, can be more cost-effective than starting from scratch. Fundamentally, the question of whether to use an EBI as is or adapt it centers on the need to define and preserve fidelity while maximizing fit to the new context (16,28). Although adaptations may compromise fidelity, they can provide gains in program adoption. Such research in health-disparity communities argues for greater attention to issues of fit within the local context at the expense of fidelity. Addressing these issues early in the planning process through dialogue between academic and community partners is imperative. Because the result is a new adapted program, it usually requires rigorous testing. As noted by Brownson and Jones (2), “If the adaptation process changes the original intervention to such an extent that the original efficacy data may no longer apply, then the program may be viewed as a new intervention under very different contextual conditions.”

In practice, translation is never straightforward, and programs evolve over time — each implementation is a slightly adapted version of the last (5). Translation involves nonlinear, iterations occurring over an extended time with adaptations throughout. For example, an EBI is adapted for a new context and vetted by stakeholders. Pretesting leads to further adaptations. Once in the field, community trials often require additional adaptations as inappropriate content or methods are discovered. As Trickett argues, clinical research usually follows a sequence in which the science is strengthened initially in the RCT (efficacy phase) by ruling out the context, while later in the effectiveness and translation phases, stakeholder involvement serves to recontextualize the scientific findings (28).

Dissemination and implementation science has several limitations that provide promising areas of research on translating behavioral interventions for individuals from vulnerable populations. The processes leading to successful adaptation and implementation in these communities and their links to health outcomes are not well measured or described (40). Most dissemination and implementation research does not account for needed adaptations to reach racial/ethnic minority, low SES, and disabled individuals. Publishing information on such adaptations, including detailed methods and results, will advance dissemination and implementation research and further the application of knowledge to promote health equity in these communities.
